# Postmortem magnetic resonance appearances of congenital high airway obstruction syndrome

**DOI:** 10.1007/s00247-014-3133-7

**Published:** 2014-09-05

**Authors:** Owen J. Arthurs, Lyn S. Chitty, Lydia Judge-Kronis, Neil J. Sebire

**Affiliations:** 1 Department of Radiology, Great Ormond Street Hospital for Children NHS Foundation Trust, London, WC1N 3JH UK; 2 Institute of Child Health, UCL, London, UK; 3 Genetics and Genomic Medicine, UCL Institute of Child Health, London, UK; 4 Great Ormond Street and UCLH NHS Foundation Trusts, London, UK; 5 Department of Histopathology, Great Ormond Street Hospital for Children NHS Foundation Trust, London, UK

**Keywords:** Congenital high airway obstruction syndrome, Magnetic resonance imaging, Autopsy, Pathology, Foetus, Child

## Abstract

**Background:**

Congenital high airway obstruction syndrome (CHAOS) is a rare life-threatening condition characterised by complete or near-complete developmental obstruction of the foetal airway. Although antenatal imaging findings have been described, the postmortem MRI findings have not been reported.

**Objective:**

To present postmortem MRI features of CHAOS.

**Materials and methods:**

We retrospectively reviewed our hospital pathology and imaging databases for cases of CHAOS over a 2-year period.

**Results:**

We identified two cases of CHAOS. In both cases, postmortem plain radiographs demonstrated gross abdominal distension with distortion and splaying of the rib cage. Both foetuses had characteristic postmortem MRI findings including large-volume fluid-filled lungs on T2-weighted imaging, diaphragmatic eversion, fluid-filled airway dilatation below the level of obstruction, centrally positioned and compressed heart, and massive ascites. One foetus had an associated limb abnormality.

**Conclusion:**

Postmortem MRI in foetuses suspected of having CHAOS allows confirmation of the diagnosis, determination of the anatomical level of the atresia or stenosis, and identification of associated abnormalities without the need for invasive autopsy.

## Introduction

Congenital high airway obstruction syndrome (CHAOS) is a rare life-threatening condition characterised by complete or near-complete intrinsic developmental obstruction of the foetal airway. It is usually fatal, and the classic features are detectable by antenatal US examination. Most cases occur sporadically, with no associated gene abnormality, although about 50% of cases have additional structural abnormalities.

Typical prenatal sonographic findings include large hyperechoic lungs with inverted diaphragm, ascites and dilated fluid-filled lower airways, and in some cases the level of the obstruction can be identified [1, 2]. It is an important diagnosis to make antenatally because planned airway management and delivery via ex utero intrapartum treatment to tracheostomy have improved the outcome in this condition, with some survivors now reported to childhood [3, 4].

Although antenatal imaging findings have been described, the postmortem MRI findings have not been reported. Postmortem confirmation of the diagnosis provides potentially important information, both for the parents and for improved understanding of the underlying pathogenesis, and hence optimisation of management for future cases [3]. However, there has been a progressive reduction in the proportion of parents consenting to standard autopsy examination, such that in the UK less than 20% of neonatal deaths undergo postmortem examination [5]. This has led to an increased interest in developing less-invasive methods of postmortem investigation, particularly using MRI in foetuses, infants and children [6]. Such non-invasive approaches appear more acceptable to both parents and health care professionals involved in the consent process, with the majority of parents agreeing to postmortem non-invasive imaging [7, 8]. Here, we present the postmortem MRI findings in two foetuses terminated for suspected CHAOS, and corresponding autopsy findings.

## Materials and methods

We retrospectively searched our hospital pathology and imaging databases for cases of CHAOS who had undergone postmortem imaging as part of the autopsy procedure. Informed consent was provided for postmortem examination and imaging, and the use of non-identifiable autopsy data for research was approved by the local research ethics committee.

Postmortem MRI is offered as part of a less-invasive autopsy service at our institution, a tertiary referral children’s hospital, to parents who decline a full autopsy. We searched our databases over the 2-year period since the introduction of postmortem imaging at our institution, 2012–2014, identifying parents of two foetuses with CHAOS who consented to both postmortem MRI and autopsy.

Before postmortem MRI, bodies were stored in a mortuary at 4°C. All scans were performed on a 1.5-T MR scanner (Avanto; Siemens Healthcare, Erlangen, Germany) using whole-body 3-D T2-weighted turbo spin echo (TSE) sequences (repetition time/echo time [TR/TE] 3,500/276 ms, voxel size 0.8 × 0.8 × 0.8 mm, 2 averages), 3-D T1-weighted volumetric interpolated breath-hold examination (VIBE) sequences (TR/TE 5.9/2.4 ms, flip angle 25°, voxel size 0.8 × 0.8 × 0.8 mm, 8 averages) and 3-D constructive interference in the steady state (CISS) sequences (TR/TE 9.2/4.6 ms, flip angle 70°, voxel size 0.6 × 0.6 × 0.6 mm, 4 averages).

Postmortem MR images were reported by a paediatric radiologist with 5 years’ experience (O.J.A.) prior to a minimally invasive, laparoscopy-assisted autopsy performed by a perinatal pathologist with more than 10 years’ experience (N.J.S.). At the time of this report, only the antenatal reports were available to the radiologist and pathologist, with antenatal imaging correlation only available after all investigations had been completed.

Laparoscopy-assisted autopsy was performed via a 30-mm lower sternal incision to allow abdominal and thoracic organ sampling [9]. The larynx was removed en bloc with the lungs for sampling. This approach allows organ examination and tissue sampling whilst limiting the extent of postmortem incisions.

## Results

### Case 1

There was no significant medical or obstetric history. Following an unremarkable initial antenatal course, routine US at about 20 weeks of gestation demonstrated a male foetus with an apparently dilated trachea with bilateral enlarged echogenic lungs, inverted diaphragm, compressed heart and severe ascites. No additional abnormalities were noted. An amniocentesis was performed for karyotyping (46 XY) and the parents opted for termination of pregnancy at 24 weeks’ gestation. Postmortem examination including postmortem MRI was offered as part of routine clinical care.

**Fig. 1:**
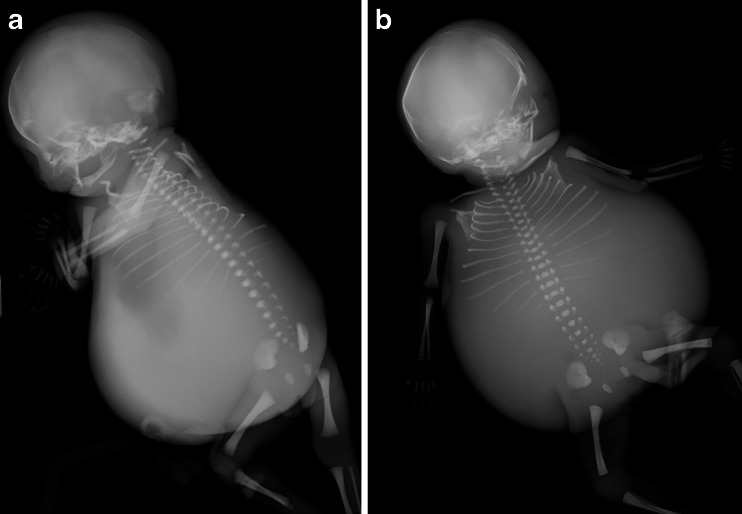
Case 1. Postmortem radiography in a 24-week male foetus with congenital high airway obstruction syndrome. **a** Lateral and (**b**) anteroposterior postmortem skeletal radiographs show gross soft-tissue swelling of the thorax and abdomen, with splaying of the ribcage. No limb anomalies were identified in this foetus

**Fig. 2:**
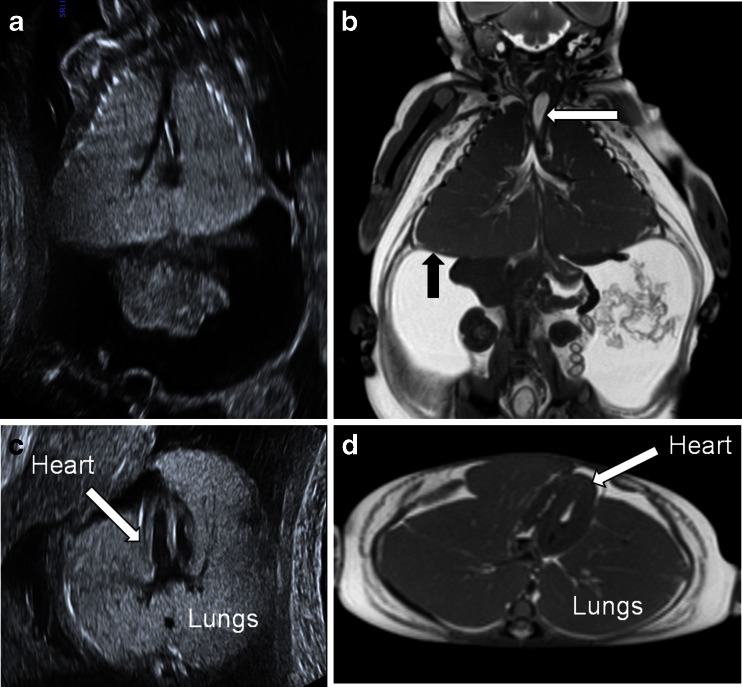
Case 1. Imaging in a male foetus (same as in Fig. 1) with congenital high airway obstruction syndrome (CHAOS), including antenatal US at 20 weeks’ gestation (**a, c**) and postmortem coronal (**b**) and axial (**d**) high-resolution T2-weighted postmortem MRI following termination at 24 weeks’ gestation. Postmortem MRI shows disproportionately gross ascites (relative to subcutaneous oedema) on coronal (**b**) and axial (**d**) abdominal imaging, marked distension of both lungs with eversion of the diaphragm and a centrally placed heart, typical features of CHAOS. The heart and lungs are indicated on axial MR image (**d**). Coronal MR image (**b**) demonstrates the fluid-filled airway (*white arrow*) below the level of the obstruction, as well as the eventration of the diaphragm (*black arrow*)

**Fig. 3:**
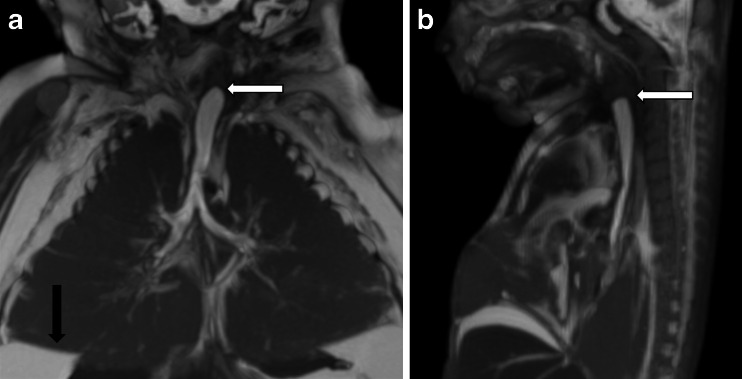
Case 1. Postmortem MRI in a male foetus, same case as in Figs. 1 and 2, with congenital high airway obstruction syndrome. Coronal (**a**) and sagittal (**b**) planar reconstructions show the dilated airway below the level of the obstruction. Note the level of the obstruction with distal fluid-filled airway (*white arrow*) and the eventration of the diaphragm (*black arrow*)

**Fig. 4:**
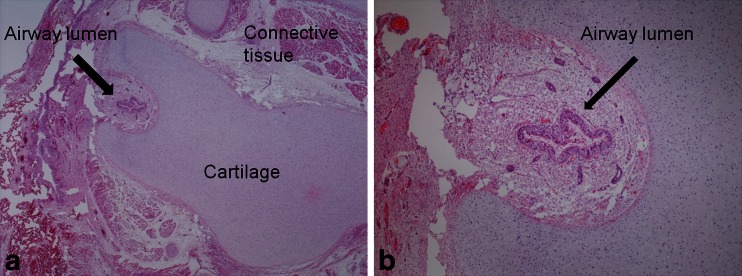
Histology of a male foetus with congenital high airway obstruction syndrome who was terminated at 24 weeks (same foetus as in Figs. 1, 2 and 3). Photomicrographs of laryngeal atresia demonstrate laryngeal cartilage with a tiny posteriorly situated lumen (**a**, haematoxylin & eosin stain, original magnification ×12.5), which on high power is lined by ciliated epithelium at this level (**b**, original magnification ×100)

Postmortem skeletal radiography showed gross soft-tissue swelling of thorax and abdomen, with splaying of the ribcage (Fig. 1) but no bone abnormality. Postmortem MRI 5 days after death showed dilatation of the trachea below the level of the obstruction, with bilateral lung expansion causing diaphragmatic eversion, corresponding to the prenatal sonographic findings (Fig. 2). The heart was centrally placed but otherwise structurally normal (Fig. 2). The abdominal organs were normal but there was gross ascites with minor septations (Fig. 2). The intracranial appearances were unremarkable. Three-dimensional multiplanar reconstructed images were used to delineate the level of the obstruction (Fig. 3). Histology is presented in (Fig. 4).

### Case 2

Following an uncomplicated initial antenatal course and low-risk screening for Down syndrome, a routine US examination at 20 weeks of gestation revealed a male foetus with bilateral enlarged echogenic lungs and ascites. An amniocentesis was carried out (46 XY). A second sonogram at a regional foetal medicine unit confirmed these findings along with generalised subcutaneous oedema, diaphragmatic eversion and bilateral talipes equinovarus. The parents opted for termination of pregnancy at 24 weeks, and postmortem examination including postmortem MRI was offered as part of routine clinical care.

**Fig. 5:**
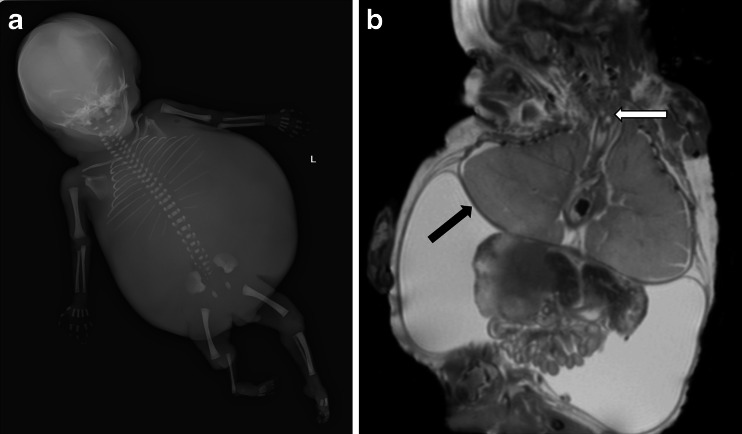
Case 2. Postmortem radiography and MRI in a male foetus with congenital high airway obstruction syndrome who was terminated at 24 weeks’ gestation. **a** Postmortem anteroposterior skeletal radiograph shows gross soft-tissue swelling of the thorax and abdomen, with splaying of the ribcage and right fibular hemimelia and severe talipes equinovarus. **b** Coronal high-resolution T2-weighted MRI 6 days after death shows similar features of disproportionately gross ascites, marked distension of both lungs with eversion of the diaphragm (*black arrow*) and a centrally placed heart. Even on sagittal high-resolution T2-weighted MR images (not shown) it was difficult to determine the level of the airway obstruction (*white arrow* in **b**)

Postmortem plain radiographs demonstrated gross abdominal distension with distortion and splaying of the rib cage, with right fibular hemimelia and talipes equinovarus (Fig. 5). The left lower limb was normal. Postmortem MRI 6 days after death showed marked ascites, distension of both lungs with eversion of the diaphragm, and a compressed centrally placed heart (Fig. 5). Generalised subcutaneous oedema was noted. Despite sagittal high-resolution T2-W MR images, the precise level of the airway obstruction could not be visualised.

In both cases, similar external appearances were noted, and laparoscopic assisted autopsy was performed including sampling of the larynx and trachea, which histologically confirmed almost complete occlusion at the level of the larynx, consistent with failure of recanalisation (Fig. 5).

## Discussion

Both foetuses had characteristic postmortem MRI findings of CHAOS, which was confirmed at autopsy. Classic imaging features included high signal intensity large-volume fluid-filled lungs on T2-W imaging, eversion of the diaphragm, airway dilatation (with fluid) below the level of obstruction, centrally positioned and compressed heart, and massive ascites as well as moderate subcutaneous oedema, and pleural and pericardial effusions. Autopsy tissue sampling allowed more specific determination of laryngeal anatomy, but this additional information did not affect patient management or counseling; the potential advantage of a postmortem MR-based approach is to offer parents an alternative to full conventional autopsy. We demonstrate that postmortem imaging, along with minimally invasive tissue sampling if required, confirms the diagnosis and might be an acceptable choice for parents who decline conventional autopsy.

Numerous reports of prenatal sonographic findings in foetuses affected by CHAOS show characteristic bilateral, enlarged and echogenic lungs associated with diaphragmatic eversion and ascites, with about 130 cases described [10–12]. In CHAOS, failure of airway canalization at 9–10 weeks of embryological development leads to upper airway obstruction. This in turn prevents appropriate clearance of fluid from the lungs during foetal life, leading to airway dilatation and progressive fluid expansion of the lungs, with pressure effect on the diaphragm and compression of the heart (and decreased venous return), leading to gross ascites [13]. The atretic airway segment can be difficult to visualise antenatally, but the tracheal dilatation is characteristic. Previous autopsy data have allowed demonstration of the microanatomy of the failure of airway recanalisation and the association between CHAOS and histologically increased lung surface area and lung volume, increased alveolar number and subjectively advanced lung maturation, providing important information regarding the pathophysiological mechanisms in this condition [14, 15].

Causes of CHAOS include laryngeal atresia, laryngeal or subglottic stenosis, webs, laryngeal cysts and tracheal atresia or stenosis. Imaging is useful to define the features of the sequence, to investigate the level of obstruction (laryngeal versus tracheal) [12, 13], and to exclude extrinsic causes such as lymphatic malformations, cervical tumours and vascular anomalies [16]. Typically the appearances are progressive, although the presence of a tracheo-oesophageal fistula or other congenital communications may limit the fluid accumulation by decreasing intra-thoracic pressure [2, 17], making antenatal diagnosis more difficult and leading to an unanticipated fatal outcome at delivery. Subcutaneous oedema and pleural and pericardial effusions are seen in CHAOS antenatally, and it can be difficult to distinguish pathological features of hydrops (associated with a worse prognosis) from normal postmortem MRI findings [18].

Although most cases of CHAOS are sporadic, about 50% of foetuses with CHAOS in other series have associated congenital abnormalities, particularly of the musculoskeletal system or digits [19]. One of our cases had a limb defect, with hemimelia and talipes equinovarus. Rarely, CHAOS is part of underlying Fraser syndrome [20], which has other characteristic anomalies (cryptophthalmos-syndactyly) and can be confirmed by mutation analysis of FRAS1.

Because they are less invasive, imaging-based methods of investigation after death are becoming increasingly important, particularly for parents who do not agree to traditional postmortem examination for moral, religious, social or other reasons [8, 10]. However, the correct interpretation of findings in this setting requires specialist knowledge of the normal variants of imaging changes that occur after death, in addition to underlying pathologies [18]. In cases such as these, where a specific body area is to be examined, full traditional autopsy might not be required to make the diagnosis, and a minimally invasive approach based on postmortem imaging is diagnostic.

One limitation of postmortem imaging in these cases is that we did not specifically adapt the imaging protocols to try to better delineate the level of the obstruction. It is possible that more advanced imaging techniques, or the installation of contrast agents (perhaps air, or gadolinium-based fluid) could delineate more precisely the level of obstruction. We thought this was unnecessary because more precise localisation of the level of obstruction has little prognostic significance. We also acknowledge that normal postmortem changes can contribute to the fluid accumulation seen in the lungs at postmortem imaging, although the contribution in this particular condition is likely to be minimal, given the close correlation between pre-mortem and postmortem imaging findings.

## Conclusion

Postmortem MRI in foetuses with suspected CHAOS allows confirmation of the diagnosis, determination of the anatomical level of the atresia/stenosis, and identification of associated abnormalities without the need for invasive autopsy.
